# Does Ability to Defend Moderate the Association between Exposure to Bullying and Symptoms of Anxiety?

**DOI:** 10.3389/fpsyg.2017.01953

**Published:** 2017-11-07

**Authors:** Morten Birkeland Nielsen, Johannes Gjerstad, Daniel Pitz Jacobsen, Ståle Valvatne Einarsen

**Affiliations:** ^1^National Institute of Occupational Health, Oslo, Norway; ^2^Department of Psychosocial Science, University of Bergen, Bergen, Norway

**Keywords:** aggression, harassment, distress, power (im-)balance, health, personality

## Abstract

In the context of workplace bullying, the ability to defend refers to whether or not a target feels able to deal with those negative behaviors that typically constitute bullying. The aim of this study was to determine whether the perceived ability to defend oneself moderates the association between exposure to bullying behaviors at work and symptoms of anxiety as predicted by the definition of workplace bullying. It was hypothesized that exposure to bullying behaviors would be more strongly related to symptoms of anxiety among targets feeling unable to defend oneself than among targets who do feel that they are able to defend themselves in the actual situation. This survey study was based on a probability sample of 1,608 Norwegian employees (response rate 32%). Only respondents exposed to at least one bullying behavior were included (*N* = 739). In contrast to hypothesis, the findings showed that ability to defend only had a protective effect on the relationship between exposure to bullying behaviors and anxiety in cases of low exposure. In cases of high exposure, there was a stronger increase in anxiety among employees able to defend themselves than among those who generally felt unable to defend. Hence, the ability to defend against exposure to bullying behaviors does not seem to protect high-exposed targets against symptoms of anxiety. Organization should therefore intervene against bullying in early stages rather than relying on the individual resilience of those exposed.

## Introduction

An extensive body of longitudinal evidence has established exposure to bullying in the workplace as a major predictor of impaired health and well-being among employees (for reviews and meta-analyses, see Nielsen and Einarsen, 2012; Nielsen et al., 2014, 2016b; Verkuil et al., 2015). Despite this interest in the individual consequences of bullying, surprisingly little is known about the variables that moderate the relationship between exposure to bullying and outcomes. Understanding moderators is highly important as it is unlikely that all targets of bullying will respond to the exposure in the same manner and to the same degree. It is far more likely that the effects of bullying are dependent upon a range of personal, situational and organizational characteristics such as personality and individual dispositions, resilience, coping strategies, social support, organizational climate, and leadership practices (Einarsen et al., 2016; Nielsen et al., 2016a).

Workplace bullying refers to the long-lasting and systematic mistreatment of an employee by other organization members. Hence, it describes a situation where an employee persistently and over a period of time, perceives to be on the receiving end of negative actions from superiors or co-workers and where the employee finds it difficult to defend him-/herself against these actions due to a real or perceived power imbalance between target and perpetrator (Olweus, 1993; Einarsen and Skogstad, 1996). Following this definition, workplace bullying takes the form a two-step process. The first step includes exposure to systematic aggression and mistreatment over time, whereas the second step comprises a power imbalance reflected through a perception of being unable to defend oneself in the actual situation (Einarsen et al., 2011; Nielsen and Knardahl, 2015). In the reminder of this article we will refer to the first step of the process as “exposure to bullying behaviors” and the second step as “ability to defend.”

Considering that powerlessness is such a central aspect of the workplace bullying phenomenon (Saunders et al., 2007; Baillien et al., 2009), it is reasonable to expect that any health and well-being outcome of being exposed to bullying behaviors are conditioned by whether the target is able to defend him-/herself against the said exposure. To elucidate the role of powerlessness in the workplace bullying process, the overarching objective of the current study was to determine whether the perceived ability to defend oneself moderates the association between exposure to bullying behaviors and symptoms of anxiety, the latter being one of the most documented outcomes of bullying (Nielsen and Einarsen, 2012; Verkuil et al., 2015). In line with well-established theories on stress (i.e., the Transactional model of stress and coping and the theory of learned helplessness) we argue that exposure to bullying is more strongly associated with anxiety among targets who feel unable to defend against the bullying behaviors than among those who feel able to defend oneself. Hence, this study extends previous literature on workplace bullying and mental health by adding to the understanding of *when* and *under what conditions* exposure to incivility and other kinds of bullying behaviors relate to the health and well-being in exposed targets.

### Ability to Defend as a Moderator – Theoretical and Empirical Evidence

In most definitions of workplace bullying, the target’s experience of powerlessness refers to a imbalance between target and perpetrator where the target is systematically exposed to mistreatment and harassment to the point where he or she has little resources to retaliate in kind (Einarsen, 1999; Samnani and Singh, 2016). Hence, targets may in varying degrees feel able to defend themselves against the unwanted behavior of the perpetrator. This power imbalance between the two parties, shaping this inability to defend oneself, can be both formal and informal in nature (Einarsen et al., 2011). Formal imbalance may occur in cases when the target is exposed to bullying behaviors from a person in a superior position in the organizational hierarchy and may therefore exist *a priori* to the bullying situation. Informal imbalance refers to cases where the source of power are mostly based on knowledge and experience, as well as access to support from influential persons (Hoel and Cooper, 2000). Informal power imbalance may also be reflected in the target’s dependence on the perpetrator(s), be it of a social, physical, economic, or psychological nature (Einarsen et al., 2011). Such powerlessness may also develop as a function of the bullying process itself as well as being a predisposition in the target (Zapf and Einarsen, 2005).

As workplace bullying by definition involves a victim-perpetrator relationship combined with a real or perceived power-imbalance between the two (Samnani and Singh, 2012), one may argue that the perceived inability to defend is a prerequisite for defining a situation as bullying. Without it the person toward whom the bullying behaviors are directed could withstand the attacks and retaliate, thus preventing the situation from further escalation (Salin, 2003a). This suggests that the ability to defend reflects some sort of individual capacity that determines whether a target can deal with the exposure to bullying behaviors and thereby also the appraisal and subsequent consequences of this exposure. Consequently, whether the target is able to defend him/herself should be a potential moderator that governs the outcomes of repeated exposure to acts of incivility and mistreatment in the workplace. To this date, this proposition has never been tested empirically.

Theoretically, the ability to defend as a potential moderator of the relation between exposure to bullying behaviors and health outcomes can be explained by well-established stress process models. In their Transactional model of stress and coping, Lazarus and Folkman (1984) proposed that the nature and severity of reactions following exposure to a given stressor are functions of a dynamic interplay between event characteristics and individual appraisal and coping processes. When a person is faced with a stressor, the person evaluates the potential threat (primary appraisal) and a judgment is made as to whether the event is positive or negative (Lazarus, 1993). As a secondary appraisal, the person evaluates how controllable the stressor is and determines whether ones available coping resources are adequate for handling and mastering the situation (Lazarus and Folkman, 1984). Consequently, following this model, the nature and severity of any outcome of bullying should be dependent upon how the target perceive the exposure to bullying behaviors and whether or not the target is able to deal with these negative acts. That is, one can expect that a target with the ability to defend intact is less influenced by the exposure compared to a target feeling unable to defend him-/herself against the said and unwanted behaviors.

Ability to defend as a moderator can also be explained by the theory of learned helplessness (Abramson et al., 1978; Abramson et al., 1980). Learned helplessness is a state of mind that may evolve when exposed to repeated and enduring painful or otherwise aversive stimuli which the targeted person is unable to escape or avoid (Maier and Seligman, 2016). This experience of being in a position in which there is no possible way to escape from harm or pain and in which an overall fatalism and resignation makes one believe that there is no point in trying to improve the situation (Nielsen et al., 2008). Extensive evidence has shown that learned helplessness is closely related to a range of health problems, including anxiety and depression (Abramson et al., 1989; Overmier, 2002). Following these principles, a target of bullying who perceives him-/herself to be unable to defend him-/herself against the bullying behaviors of a given perpetrator should be more likely to resign into a situation of helplessness which then may lead to increased mental distress, in our case anxiousness.

While there are no previous studies that have explicitly examined the ability to defend as a potential moderator, some findings exist on individual dispositions that may reflect the ability to defend. As noted above, the Transactional Model of Stress and coping highlights a secondary appraisal where the focal person evaluates how controllable the said stressor is thereafter determines whether ones available coping resources are adequate for handling and mastering the situation (Lazarus and Folkman, 1984). Hence, once perceived coping resources may be especially relevant with regard to the perceived ability to defend. In a review of the literature on the use of coping, the coping method that appeared to consistently produce a significant improvement in a victim’s conditions was finding a way to avoid the perpetrator(s) or to leave the situation (Aquino and Thau, 2009). Similarly, in a study of 224 Danish workers, Mikkelsen and Einarsen (2002b) found that generalized self-efficacy moderated the relationship between exposure to bullying and psychological health complaints, thus indicating that employees who have a strong belief in their own general abilities to handle problems, have a lower risk of reporting health complaints. However, other studies have provided non-significant (Nielsen et al., 2013b) or contradicting findings (Nielsen et al., 2008; Vie et al., 2011; Hewett et al., 2016), something that highlights the need for further research on such individual factors as potential moderators.

### Aims of the Study and Hypothesis

It has previously been established that exposure to bullying in the workplace is associated with increased psychological distress (Nielsen and Einarsen, 2012; Nielsen et al., 2014; Verkuil et al., 2015). There is, however, a shortage of evidence on factors that determines when bullying behaviors is associated with distress. Perceived ability to defend is a central aspect in the very definition of workplace bullying and personal capacities are highlighted as buffering factors in both the Transactional model of stress and coping and the theory of learned helplessness. Consequently, there are strong theoretical reasons for expecting a protective effect with regard to the health outcomes of exposure to bullying. To add to the understanding of the role of the ability to defend in the bullying process the overarching aim of this study was to investigate whether the ability to defend moderates the relationship between exposure to bullying behaviors and symptoms of anxiety. Based on the abovementioned theoretical models, we expect that ability to defend has a protective effect on anxiety, leading us to put forward the following hypothesis to be tested:

H1: Perceived ability to defend oneself against exposure to bullying behaviors moderates the association between exposure and symptoms of anxiety, so that the relationship is stronger for targets who are unable to defend themselves as compared to targets who are able to defend themselves against these behaviors.

## Materials and Methods

### Design and Sample

This study is based on a survey of the Norwegian working force where a random sample of 5000 employees was drawn from The Norwegian Central Employee Register by Statistics Norway (SSB). The Norwegian Central Employee Register is the official register of all Norwegian employees, as reported by employers. Criteria for sampling were adults between 18 and 60 years of age employed in a Norwegian enterprise. Questionnaires were distributed through the Norwegian Postal Service during the spring 2015, with a response rate of 32 percent. Altogether 1,608 questionnaires were satisfactory completed and included in this study. The survey was approved by the Regional Committee for Medical Research Ethics for Eastern Norway. Responses were treated anonymously, and informed consent was given by the respondents. The procedure for this study has previously been described elsewhere (Nielsen et al., 2017).

As the overarching aim of this study was to examine the interaction between exposure to bullying behaviors and ability to defend, the sample was limited to respondents who reported exposure to at least one bullying behaviors in the employed Negative Acts Questionnaire Revised (*N* = 739). Mean age in this final sample was 43.98 (*SD* = 10.28) years with a range from 21 to 61. The gender distribution was 51.4% women and 48.6% men. In total, 47.3% were married, 28.7% were common-law partner, 15.3% were unmarried, and 8.6% were widowed, separated, or divorced. Altogether 7.7% had less than 11 years of education, 33.4% had between 11 and 13 years, 31.6% had between 14 and 17 years, while 27.3% had 18 years or more. A total of 88.1% were in a full-time employment, 6% in part time employment and 5.3% were on a sick leave or occupational rehabilitation, whereas 0.6% was disabled pensioners or retired. Altogether 36.4% had a leadership position with personnel responsibilities.

### Instruments

Exposure to bullying behaviors in the workplace was measured with the 9-item version of the *Negative Acts Questionnaire – Revised* (NAQ-R) inventory (Einarsen et al., 2009). NAQ-R describes negative and unwanted behaviors that may be perceived as bullying if occurring on a regular basis. All items are formulated in behavioral terms and hence focus on the mere exposure to inappropriate behaviors while at work with no references to the term bullying (Einarsen and Nielsen, 2015). The NAQ-R contains items referring to both direct (e.g., openly attacking the victim) and indirect (e.g., social isolation, slander) behaviors (Einarsen et al., 2009). The items do also distinguish between personal and work related forms of bullying (Einarsen et al., 2009). The respondents were asked to indicate how often they had been exposed to each specific item in questionnaire at their present worksite during the last 6 months. Response categories range from 1 to 5 (‘never,’ ‘now and then,’ ‘monthly,’ ‘weekly’ and ‘daily’). This nine item version of the NAQ-R had a Cronbach’s alpha of 0.81 in this study.

*Ability to defend* was measured with a single item question developed specifically for this study. The item follows the self-labeling method for assessing workplace bullying and is based on the part of the definition of workplace bullying that describes the power imbalance between the target and perpetrator (Nielsen et al., 2011). Directly following the NAQ-R, the respondents were asked “If you have been exposed to one or more of the behaviors in the list above, did you find it difficult to defend yourself against this exposure? Response alternatives were “Not exposed to any of the acts,” “No, never,” “Yes, once in a while,” “Yes, often.”

Self-labeled victimization from workplace bullying was measured with the well-established self-labeling method (Olweus, 1991; Einarsen and Skogstad, 1996; Solberg and Olweus, 2003; Nielsen et al., 2011). After being presented with the following definition: “*Bullying (harassment, badgering, niggling, freezing out, offending someone) is a problem in some workplaces and for some workers. To label something bullying it has to occur repeatedly over a period of time, and the person confronted has to have difficulties defending himself/herself. It is not bullying if two parties of approximately equal “strength” are in conflict or the incident is an isolated event*” (Einarsen and Skogstad, 1996, p. 191), respondents were asked “*Have you been subjected to bullying at the workplace during the last 6 months?*” The response categories were “no,” “rarely,” “now and then,” “once a week,” and “several times a week.”

*Symptoms of anxiety* during the last week were measured by five items measuring typical symptoms of anxiety from the anxiety subscale in the Hopkins Symptom Checklist (HSCL-25). The HSCL is a valid and reliable (Rickels et al., 1976) self-administered instrument measuring mental distress (anxiety, depression, and psychosomatic complaints) in population surveys (Derogatis et al., 1974). Comparisons have found that shorter versions perform as well as the more extensive versions of the inventory (Strand et al., 2003). Responses were given on a four-point scale, ranging from “1 = not at all” to “4 = extremely.” Example items are “Heart pounding or racing” and “Feeling fearful.” Cronbach’s alpha for this scale was 0.73 in the current study.

### Control Variables

The following control variables were included in the study: Age, seniority at current workplace, gender, leadership responsibility, and full-time vs. other forms of employment. Although existing evidence is inconclusive, studies have established age differences in workplace bullying (De Cuyper et al., 2009). As for gender, findings show gender differences in prevalence of bullying, (Björkqvist et al., 1994; Salin, 2003b), outcomes of bullying (Rodriguez-Munoz et al., 2010; Einarsen and Nielsen, 2015; Attell et al., 2017), and ways of coping with bullying (Ólafsson and Jóhannsdóttir, 2004). Seniority at current workplace was included as a control variable since workplace bullying by definition is a long-lasting form of exposure. Respondents with relatively short seniority may therefore be less likely to perceive potential exposure as bullying. Power imbalance is another defining aspect of bullying. To account for formal power imbalance, we adjusted for whether or not the respondents had a leadership position at the workplace. Finally, as persons with a full-time position spend more time at the workplace, and therefore should have a higher risk of negative social interactions, compared to employees with part-time employment, we adjusted for employment status.

### Statistical Analyses

Statistical analyses were conducted with IBM SPSS 24.0. The level of significance was set to *p* < 0.05. For all measurement inventories, summary scales were calculated on the basis of a mean-score of their respective items. Missing data in scale variables were replaced with the Hot Deck imputation procedure (Myers, 2011). This method handles missing data by substituting each missing value with an observed response from a respondent with similar characteristic. Age, gender, and leadership position were used as predefined anchor variables in the imputation procedure.

To explore main and moderating effects, we conducted a hierarchical regression analysis, to test for linear associations between exposure to bullying behaviors and symptoms of anxiety, as well as the interactive effects of exposure to bullying and the ability to defend, with regard to anxiety. The guidelines by Baron and Kenny (1986) were followed, and, in line with Aiken and West (1991), the continuous predictor variables were centered prior to the two-way interaction analysis. The SPSS macro “Interaction and simple slopes test with one continuous and one dichotomous variable” by Jason T. Newsom^1^ was used to generate the regression estimates, plots, and simple slopes analyses.

## Results

### Validity of the “Ability to Defend” Measure

The indicator of whether the respondents were able to defend themselves against exposure to bullying behavior is a newly developed measure that has not been included in any previous studies. To provide indications of its validity, the measure was therefore compared with other measures of workplace bullying. A Spearman correlation analyses showed a significant correlation of 0.34 (*p* < 0.001) between self-labeled victimization from workplace bullying and ability to defend thus indicating an overlap between the indicators and in line with major definitions of workplace bullying. As the measure of ability to defend is limited to only one aspect of workplace bullying, whereas the questions about victimization should tap all four definitional aspects (i.e., negative acts, repetition, duration, and power imbalance), a correlation of 0.34 seems reasonable. A Spearman correlation of 0.50 (*p* < 0.001) was established between ability to defend and exposure to bullying behaviors as measured by the NAQ. A follow-up one-way ANOVA showed that respondents in the “Yes, once in a while” (*M* = 1.48; *SD* = 0.35) and “Yes, often” (*M* = 2.14; *SD* = 0.79) categories reported significantly (*F* = 167.50; df = 2/734; *p* < 0.001) higher exposure to bullying behaviors compared to the “No, never” (*M* = 1.25; *SD* = 0.22) category of the ability to defend indicator. As it should be harder to defend against bullying with increasing exposure, this finding is in line with reasonable expectations.

Due to few cases in the “Yes, often” category (*n* = 46), there was insufficient statistical power to examine moderating effects with all three response categories of the ability to defend measure. Hence, positive responses (i.e., “Yes, once in a while” and “Yes, often”) were recoded into a single category in the main correlation and regression analyses. This is in line with previous studies that have used dichotomized single item measures to assess aspects of workplace bullying (Hauge et al., 2007; Nielsen et al., 2011).

### Descriptive Findings

Means, standard deviations, and intercorrelations for all study variables are displayed in **Table 1**. Altogether 42% of the respondents who reported exposure to at least one bullying behaviors felt unable to defend themselves against the exposure. Mean scores and standard deviations for exposure to bullying behaviors and anxiety were rather small, thus indicating relatively low exposure and variance in the sample. Exposure to bullying behaviors were positively correlated with symptoms of anxiety (*r* = 0.29; *p* < 0.001) and the ability to defend (*r* = 0.40; *p* < 0.001). Ability to defend was positively associated with anxiety (*r* = 0.21; *p* < 0.001).

**Table 1:** Frequencies, means, standard deviations (SD) and intercorrelations for study variables (*N* = 737).

	Variable	%	*M*	*SD*	1	2	3	4	5	6	7	8
1	Gender (women)	51%	–	–	–							
2	Leadership responsibility	36%	–	–	-0.23^∗∗∗^	–						
3	Full-time employment	88%	–	–	0.15^∗∗∗^	-0.12^∗∗∗^	–					
4	Age	–	43.98	10.28	-0.05	0.11	-0.02	–				
5	Seniority at current workplace	–	10.84	9.12	-00.10^∗∗^	0.10^∗∗^	-0.03	0.46^∗∗∗^	–			
6	Exposure to bullying behaviors	–	1.39	0.40	-0.03	-0.02	0.03	-0.01	-0.06	**0.81**		
7	Anxiety	–	1.43	0.42	-0.06	-0.05	0.15^∗∗∗^	-0.10^∗∗^	-0.07^∗^	0.29^∗∗∗^	**0.73**	
8	Ability to defend	42%	–	–	0.11^∗∗^	-0.09^∗^	0.08^∗^	-0.01	-0.04	0.40^∗∗∗^	0.21^∗∗^	–

*Cronbach’s alpha in bold along the diagonal. ^∗^*p* < 0.05; ^∗∗^*p* < 0.01; ^∗∗∗^*p* < 0.001.*

### Main and Interaction Effects

Findings from the multiple regression analyses of linear associations and interaction effects are presented in **Table 2**. For the linear association, the control variables age, gender, seniority, employment status, and leadership position explained four percent of the variance in anxiety (*R^2^* = 0.04; *p <* 0.001; *F* = 5.23; *df* = 5/730; *p <* 0.001). Being in a full-time employment (*β* = 0.15; *p* < 0.001) was the only significant control variable. The explained variance in anxiety increased to 12% (*R^2^* = 0.12; *p* < 0.001) when exposure to bullying behaviors (*β* = 0.24; *p* < 0.001) and the ability to defend (*β* = 0.10; *p <* 0.05) was included in the model (*F* = 14.30; *df* = 7/728; *p <* 0.001). The amount of explained variance increased significantly by one percent when the interaction term was added to the regression (*R^2^* = 0.13; *p* < 0.001; Δ*R^2^* = 0.01; *p* < 0.05). The interaction term made a significant contribution to the explained variance (*β* = -0.16; *p <* 0.05), and the interaction model was significant (*F* = 13.09; *df* = 8/727; *p <* 0.001). This means that there is an interaction effect between exposure to bullying behaviors and the ability to defend against these acts with regard to symptoms of anxiety.

**Table 2:** Main and interactive effects of exposure to bullying behaviors and ability to defend on symptoms of anxiety (*N* = 737).

Step	Variable	B	SE B	*β*	*R*^2^	Δ*R*^2^
1					0.04	
	Age	-0.00	0.00	-0.08		
	Seniority	-0.01	0.00	-0.03		
	Gender (reference category: “Male”)	0.02	0.03	0.02		
	Leadership responsibility (reference category: “No”)	-0.02	0.03	-0.02		
	Full-time employment (reference category: “No”)	0.19	0.05	0.15***		
2					0.12	0.08
	Age	-0.00	0.00	-0.09*		
	Seniority	0.00	0.00	-0.01		
	Gender	0.02	0.03	0.04		
	Leadership responsibility	-0.01	0.03	-0.01		
	Full-time employment	0.17	0.05	0.13***		
	Bullying behaviors (Bullying)	0.25	0.04	0.24***		
	Ability to defend (AtD)	0.08	0.03	0.10*		
3					0.13	0.01^∗^
	Age	-0.00	0.00	0.08*		
	Seniority	-0.00	0.00	0.01		
	Gender	0.03	0.03	0.04		
	Leadership responsibility	-0.00	0.03	0.04		
	Full-time employment	0.17	0.05	0.13***		
	Bullying	0.41	0.09	0.39***		
	AtD	0.07	0.03	0.08*		
	Interaction term: Bullying^∗^AtD	-0.19	0.10	-0.16*		

*^∗^*p* < 0.05; ^∗∗^*p* < 0.01; ^∗∗∗^*p* < 0.001.*

**FIGURE 1:**
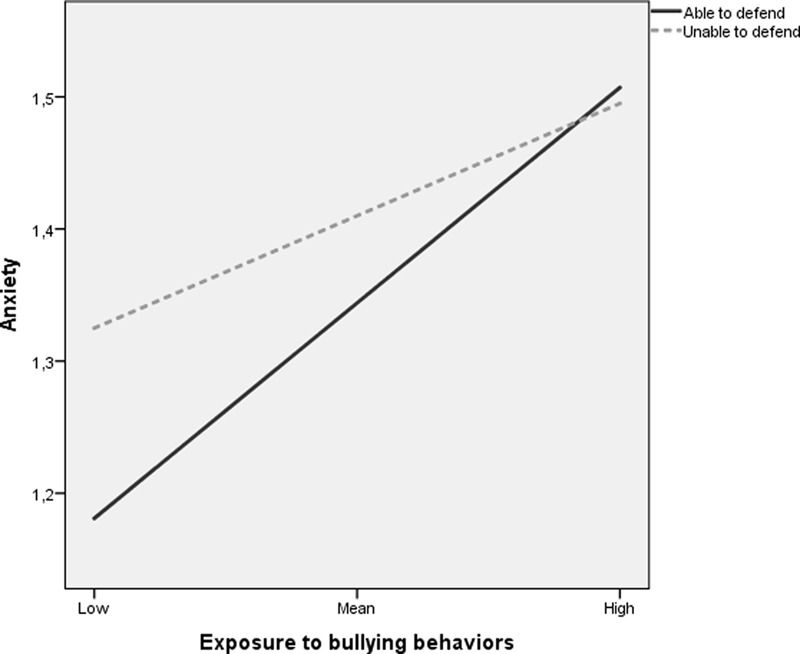
Interactive effect of exposure to bullying behaviors and ability to defend on symptoms of anxiety.

To examine the nature of this interaction, scores were plotted at the mean, low (1 *SD* below the mean) and high (1 *SD* above the mean) values on the indicator of bullying behaviors and for each of the two categories of the ability to defend measure. As shown in **Figure 1**, the results indicate a stronger relationship between exposure to bullying behaviors and symptoms of anxiety for targets *with* the ability to defend themselves against the bullying when compared to those who perceived themselves to be unable to defend against these acts. Follow-up analyses of simple slopes confirm this interpretation by revealing that exposure to bullying behaviors were more strongly related to symptoms of anxiety among targets who reported to be able to defend themselves (*β* = 0.39; *p* < 0.001) than among targets being unable to defend (*β* = 0.20; *p* < 0.001). The Ratio of Residual Variances in the two groups was 0.77. A ratio value between 0.67 and 1.5 does not violate homogeneity assumptions (DeSchon and Alexander, 1996). In direct contrast to our study hypothesis about a protective effect of being able to defend oneself against bullying, the results indicate a “reverse buffer association” where ability to defend oneself seems to only have a protective effect on the relationship between bullying behaviors and anxiety in cases of low exposure to bullying behaviors. When exposure to bullying behaviors is high, there was a stronger increase in levels of anxiety among respondents with ability to defend as compared to respondents unable to defend.

The findings remained consistent when the regression analyses were repeated without including control variables.

## Discussion

The aim of this study was to determine the impact of being able to defend oneself on the already well-established association between exposure to bullying behaviors and symptoms of anxiety. It was expected that targets with an intact ability to defend themselves against exposure to bullying behaviors would have lower levels of anxiety compared to targets that were unable to defend. In direct contrast to the hypothesis, the findings showed that ability to defend only had a protective effect against anxiety in cases of low exposure to bullying behaviors. In cases of high exposure, targets with ability to defend reported equally high levels of anxiety as targets without this ability. This finding is not only in contrast to the study hypothesis, but also to the theoretical models which constituted the background for our expectations of the ability to defend as a moderator, that is the Transactional theory of stress and coping and the theory on learned helplessness.

Although this finding goes against theoretical assumptions about individual capacities as protective resources, it is in line with some previous studies on workplace aggression showing that the moderating effect of individual factors is dependent upon the intensity of the exposure (Nielsen et al., 2008; Ilies et al., 2011; Britton et al., 2012; Reknes et al., 2016). For instance, both Vie et al. (2011) and Hewett et al. (2016) found that self-labeling as a victim of bullying (using a self-labeling question based on a definition of bullying where the ability to defend is a central aspect), influenced the impact of exposure to bullying on the targets’ health in cases of low exposure to bullying behaviors only. When facing intense bullying behaviors, exposure to bullying was as strongly associated with health complaints among those who did and did not self-label as a victim of bullying. Similarly, in a longitudinal study of 1582 Norwegian nurses which examined coping styles as moderators of the association between exposure to bullying and subsequent anxiety, it was found that active goal-oriented coping was only beneficial when exposure to bullying was low (Reknes et al., 2016). The effect diminished as the bullying intensified, something that suggests that high exposure to bullying behaviors has negative consequences for targeted employees regardless of their coping style. Systematic and ongoing exposure to bullying behaviors is something most people will have difficulties defending against and cope with irrespective of any coping resources they may poses (see Zapf and Einarsen, 2005 for a discussion).

The behavioral concordance hypothesis suggest that individuals experience negative affect when they engage in behaviors that are contrary to their nature (Ilies et al., 2011). An experience of situational incongruence may therefore be possible explanations for the finding of a reverse buffering effect of ability to defend on the relationship between bullying behaviors and anxiety. Building on a person-environment fit perspective, the situational-congruence model proposes that a person will experience more positive and less negative affect when there is congruence between a given situation and personality (Pervin, 1993). In contrast, individuals will experience heightened negative affect in situations that are incompatible with their personality characteristics (Diener et al., 1984; Ilies et al., 2011). With regard to workplace bullying and the ability to defend, it is therefore likely that anxiety will emerge as a response when the individual experience an incongruence between self-concept (“I am able to defend myself”) and external exposures (exposure to bullying behaviors) as this creates an imbalance between the targets own perception of him-/herself and actual life experiences.

An implication of situational incongruence is that the effects of exposure to bullying may be more prominent when interpreted against a backdrop of a positive view of oneself and the world (Nielsen et al., 2008). Consequently, for a target with an overall pervasive and enduring feeling of confidence displayed through a perception of being able to defend oneself, long-lasting and systematic exposure to severe forms of bullying may have especially negative effects because it is unanticipated and creates a pervasive feeling of dissonance in the target. Thus, being repeatedly exposed to bullying over a long period of time may result in an incongruity between the self-perception of persons able to defend themselves and how they feel they are treated by the bullies (Nielsen et al., 2008). As we need consistency in our conceptual system, such unresolved incongruence may be experienced as deeply shattering and may consequently result in psychological distress (Janoff-Bulman, 1992; Mikkelsen and Einarsen, 2002a).

The findings of a reverse buffering effect of ability to defend may also be explained by the very nature of workplace bullying as a stressor (Zapf and Einarsen, 2005). Unlike exposure to other stressors encountered at work such as job demands and role stressors, “…the aggressive behavior experienced by targets of bullying is likely to thwart the satisfaction of fundamental psychological and relational needs (e.g., sense of belonging and trust in others) and thereby inflict severe psychological, emotional, and even physical reactions” (Hauge et al., 2010, p. 427). Bullying is a particularly strong stressor that by its very nature is difficult to defend against, particularly at the workplace where fleeing or avoiding the situation is not really an option, at least in the short run. In addition, bullying is a one-sided event where the target per definition is unable to control the situation. In line with the findings of the current study, this may imply that perceived ability to defend may have a ceiling effect, being beneficial under exposure to milder forms of bullying (e.g., more like incivility, see Hershcovis et al., 2017), whereas the ability to defend does not protect targeted employees in cases of systematic harassment.

### Strengths and Limitations

In terms of strengths, the present study is based on a large and randomly selected sample of Norwegian employees. Both exposure to workplace bullying and anxiety were assessed with well-established and psychometrically sound measurement instruments. With 32% response, the overall response rate was lower than the average rate of 52% which has been established for survey research (Baruch and Holtom, 2008). Yet, while response rate has important implication for the external validity (i.e., generalization) of studies, it can be questioned whether it has any significant impact on the internal validity of a study (Schalm and Kelloway, 2001; Nielsen and Knardahl, 2016).

As all cross-sectional questionnaire surveys, our study does not account for the causal relationships between the study variables. Although we have investigated the theoretical assumption that the ability to defend moderates the relationship between exposure to workplace bullying behaviors as predictor variable and anxiety as outcome variable (see Reknes et al., 2014 for evidence), other kinds of relationships are also likely. For instance, some prospective studies have shown that the association between bullying and mental health is bidirectional (Nielsen and Einarsen, 2012; Nielsen et al., 2014; Verkuil et al., 2015). To provide indications of causality, longitudinal studies on bullying, ability to defend, and anxiety are needed.

All data were collected using self-report questionnaires, something which could hamper the internal validity of the findings. For instance, there is the possibility of common method variance and response set tendencies (Spector, 2006). Social desirability may be a likely form of response set. Social desirability is a form of response bias where the respondents answer questions in a manner that will be viewed favorably by others. It can either be over-reporting “good behavior” or under-reporting “bad,” or undesirable behavior (Phillips and Clancy, 1972). Relying on self-report methodology may be especially problematic with regard to assessing workplace bullying, ability to defend, and anxiety due to feelings of shame and guilt among respondents (Hauge et al., 2009). Yet, one may also argue that self-report is the only valid measure of these particular individual and psychological states.

The respondents’ ability to defend was measured with a single item developed specifically for this study. The use of single-item measures is often discouraged from a psychometric point of view as such measures may suffer from reliability and validity issues (Nielsen et al., 2013a). This rigorous view of single-item measures has recently been challenged (Wanous and Reichers, 1996; Wanous et al., 1997; Gardner et al., 1998). As highlighted by Olsen et al. (2012), single-item measures can be reliable, as estimated by test-retest correlations (Littman et al., 2006), correlate strongly with multiple-item scales (Wanous et al., 1997), and can predict outcomes effectively (e.g., Nagy, 2002). While single-item measures have limitations, they do also have clear advantages, such as cost-efficiency, greater face validity, and the increased willingness of respondents to take time to complete the questionnaire when the number of items is reduced (Olsen et al., 2012). The single-item method used in this study was found to correlated adequately with the most frequently used indicators of workplace bullying (self-labeling method and behavioral checklist) and do thereby seem to be a valid and reliable indicator of ability to defend. Nonetheless, to further elucidate the impact of ability to defend, a scale instrument should be developed for future studies.

## Conclusion, Implications, and Further Research

The present study showed that the perceived ability to defend oneself against workplace bullying behavior is only protective against symptoms of anxiety in cases of low exposure. This protective effect diminishes in cases where the bullying is more systematic and severe. Specifically, in cases of high exposure to bullying, there was a stronger increase in anxiety among employees able to defend themselves than among those felt unable to defend. As for relative levels of anxiety, the findings suggest that in cases of high exposure to bullying, targets report equal levels of anxiety irrespectively of their ability to defend. Hence, adhering to some previous studies (Vie et al., 2011; Nielsen et al., 2008; Reknes et al., 2016), our study further demonstrate that bullying is a detrimental experience for all those exposed, irrespective of their personal coping resources.

This finding has several important implications, be it for theory, for practice and for methodology. With regard to theory, it has been argued that although all interaction types have the potential for advancing theory, “the buffering interactions hold the greatest potential because they are more likely to challenge existing perspectives” (Andersson et al., 2014, p. 1068). In line with this claim, the reverse buffering interaction established in this study questions a central assumption in stress theory, namely that personal dispositions and personal resources will act to protect individuals against the potential negative impact of stressors. If our findings can be validated in upcoming research, preferably with designs that allows for causal interpretation (see Ilies et al., 2011; Reknes et al., 2016), stress theories must take into consideration that the protective power of personal resources may be dependent upon type of stressor rather than solely assuming that personal factors buffers the negative impact of all stressors. Alternatively, the established reverse buffering effect may suggest that personal resources have some sort of ceiling effect with regard to bullying in that they are only beneficial under low exposure.

The results of this study may also have important implications for the understanding of workplace bullying as a phenomenon. As most definitions highlight power imbalance as a main characteristic, the results of this study suggest that bullying may be detrimental even when targets perceives to have the ability to defend themselves against the mistreatment. Hence, our findings indicate that it is the very magnitude and frequency of the exposure that constitutes the menace rather than the perceived power differences between target and perpetrator. Alternatively, it may be that the power imbalance is actually manifested in the very exposure, and that this imbalance has a more profound impact on the target as compared to the subjective perception of being able to defend oneself. Nonetheless, as our study only represents a single contribution to the field, further research is needed in order to comprehend the impact of bullying on its targets.

As for methodology, the present study indicates that behavioral checklists such as the NAQ, are valid measures of workplace bullying even if not explicitly measuring all aspects of the theoretical definition, in this case not explicitly measuring the ability to defend oneself in the actual situation. With regard to practice, knowledge about factors that protects workers against workplace bullying is highly important for both managers, consultants, counselors and medical personnel. The relationships between bullying, ability to defend, and anxiety found in this study provide organizations and practitioners with important information about how to prevent and handle bullying. It has been proposed that organizations could use personality testing to identify potential targets and thereby to focus anti-victimization interventions at the identified individuals and their workplaces (Bowling et al., 2010). In light of previous studies which have found that bullying impacts all, irrespective of their personality characteristics (Nielsen et al., 2008; Reknes et al., 2016), our findings indicate that individual capacities have little protective impact with regard to bullying. Hence, it can be discussed whether testing and identifying individuals with specific personality characteristics have any merit.

As being exposed to bullying may be experienced as particularly devastating and harmful by the presumably robust employee due to incongruence and dissonance, organizations and employers must actively intervene in the early stages of the bullying process rather than believing that the said targeted worker should be able to deal with the exposure him-/herself. Previous research have shown that organizational factors, such as climate for conflict management, may be especially valuable with regard to managing workplace bullying (Einarsen et al., 2016). Consequently, focusing on primary interventions, such as building a strong psychosocial safety climate may be the most effective way to prevent workplace bullying from occurring and harming employees (Bond et al., 2010; Law et al., 2011). However, in cases where bullying does occur, organizations must have effective, and preferably pre-defined, secondary and tertiary intervention strategies in place.

## Ethics Statement

The study was approved by the Regional Committees for Medical and Health Research Ethics (REK) for eastern Norway and was conducted in accordance with the World Medical Association Declaration of Helsinki. All study participants provided were provided information that informed consent was given by answering the questionnaire. Data were de-identified for analyses. The consent procedure was approved by the REK.

## Author Contributions

All authors contributed to the development of this study and the writing of the manuscript. MN was responsible for the data analyses and the first draft of the paper. JG, DJ, and SE contributed to the writing and provided quality checks of data analyses.

## Conflict of Interest Statement

The authors declare that the research was conducted in the absence of any commercial or financial relationships that could be construed as a potential conflict of interest.

## Footnotes

1 http://web.pdx.edu/~newsomj/
